# Temporal trends, predictors, and outcomes of acute kidney injury and hemodialysis use in acute myocardial infarction-related cardiogenic shock

**DOI:** 10.1371/journal.pone.0222894

**Published:** 2019-09-18

**Authors:** Saraschandra Vallabhajosyula, Shannon M. Dunlay, Gregory W. Barsness, Saarwaani Vallabhajosyula, Shashaank Vallabhajosyula, Pranathi R. Sundaragiri, Bernard J. Gersh, Allan S. Jaffe, Kianoush Kashani

**Affiliations:** 1 Department of Cardiovascular Medicine, Mayo Clinic, Rochester, Minnesota, United states of America; 2 Division of Pulmonary and Critical Care Medicine, Department of Medicine, Mayo Clinic, Rochester, Minnesota, United states of America; 3 Department of Health Science Research, Mayo Clinic, Rochester, Minnesota, United states of America; 4 Division of Hospital Internal Medicine, Department of Medicine, Mayo Clinic, Rochester, Minnesota, United states of America; 5 Division of Nephrology and Hypertension, Department of Medicine, Mayo Clinic, Rochester, Minnesota, United states of America; Azienda Ospedaliero Universitaria Careggi, ITALY

## Abstract

**Background:**

There are limited data on acute kidney injury (AKI) complicating acute myocardial infarction with cardiogenic shock (AMI-CS). This study sought to evaluate 15-year national prevalence, temporal trends and outcomes of AKI with no need for hemodialysis (AKI-ND) and requiring hemodialysis (AKI-D) following AMI-CS.

**Methods:**

This was a retrospective cohort study from 2000–2014 from the National Inpatient Sample (20% stratified sample of all community hospitals in the United States). Adult patients (>18 years) admitted with a primary diagnosis of AMI and secondary diagnosis of CS were included. The primary outcome was in-hospital mortality in cohorts with no AKI, AKI-ND, and AKI-D. Secondary outcomes included predictors, resource utilization and disposition.

**Results:**

During this 15-year period, 440,257 admissions for AMI-CS were included, with AKI in 155,610 (35.3%) and hemodialysis use in 14,950 (3.4%). Older age, black race, non-private insurance, higher comorbidity, organ failure, and use of cardiac and non-cardiac organ support were associated with the AKI development and hemodialysis use. There was a 2.6-fold higher adjusted risk of developing AKI in 2014 compared to 2000. Presence of AKI-ND and AKI-D was associated with a 1.3 and 1.7-fold higher adjusted risk of mortality. Compared to the cohort without AKI, AKI-ND and AKI-D were associated with longer length of stay (9±10, 12±13, and 18±19 days respectively; p<0.001) and higher hospitalization costs ($101,859±116,204, $159,804±190,766, and $265,875 ± 254,919 respectively; p<0.001).

**Conclusion:**

AKI-ND and AKI-D are associated with higher in-hospital mortality and resource utilization in AMI-CS.

## Introduction

Acute myocardial infarction (AMI) continues to remain a leading cause of death worldwide, and in patients with concomitant cardiogenic shock (CS), the mortality is nearly 10-times higher.[1–11] In AMI-CS, there is often non-cardiac organ failure–neurological, respiratory, renal, hepatic and hematological. [5, 6, 11–13] Acute kidney injury AKI) is a challenging public health epidemic and is associated with high healthcare utilization.[14, 15] AKI with no need for dialysis (AKI-ND) and AKI requiring dialysis (AKI-D) are associated with worse mortality and higher resource utilization in patients with septic shock and smaller studies on AMI. [12, 13, 16–18] However, there are limited large-scale data on the prevalence of AKI-ND and AKI-D in admissions with CS the United States. Previously, in an undifferentiated national cohort of CS patients, Adegbala et al. demonstrated AKI to be associated with higher in-hospital mortality and resource usage; however this was not specific to AMI patients.[19] Using a large, nationally-representative database, we sought to assess 15-year national temporal trends of AKI in AMI-CS. We hypothesized that patients with AMI-CS have evolved into a more complex population with greater incidence and intensity of AKI, including AKI-D during this 15-year study period.

## Material and methods

The Healthcare Cost and Utilization Project-National Inpatient Sample (HCUP-NIS) is the largest all-payer database of hospital inpatient admissions (20% stratified sample).[20] Admissions from January 1, 2000 to December 31, 2014 with a primary diagnosis of AMI (International Classification of Diseases 9 Clinical Modification [ICD-9CM] 410.x) and concomitant CS (ICD-9CM 785.51) without maintenance dialysis (ICD-9CM V45.11) were included. Prior hemodialysis use was identified in 3,996 (0.9%) and these admissions were excluded. AKI was identified using ICD-9CM 584 (acute renal failure (ARF), 584.5 (ARF with tubular necrosis), 584.6 (ARF with renal cortical necrosis), 584.7 (ARF with papillary necrosis), 584.8 (ARF with other pathological lesion), and 584.9 (ARF, unspecified), which has been shown to have a high specificity (98%) and negative predictive value (96%).[21] AKI-D was identified using ICD-9CM 39.95 with AKI codes, (94% positive predictive value and 96.1% negative predictive value).[21] Using previously employed methodology, demographics, hospital characteristics, comorbidities, hospitalization diagnoses and procedures (**Table A in** S1 File) were identified for individual admissions.[2–6, 9–11, 22, 23] The primary end-point was in-hospital mortality in AMI-CS with no AKI, AKI-ND, and AKI-D. Secondary end-points included the temporal trends, length of stay, hospitalization costs and discharge disposition in admissions with no AKI, AKI-ND, and AKI-D.

### Statistical analysis

Trend weights provided by the HCUP-NIS were used to re-weight the data to adjust for the 2012 HCUP-NIS re-design. Using trend weights available on the HCUP-NIS database, samples from 2000–2011 were retroactively re-weighted. The new sampling strategy is expected to result in more precise estimates than the previous HCUP-NIS design by reducing sampling error. Chi-square and t-tests were used to compare categorical and continuous variables respectively. Data were presented as odds ratio (OR) and 95% confidence intervals (CI). Logistic regression was used to analyze the temporal trends after adjustment for confounders. Multivariable hierarchical logistic regression analysis incorporating age, gender, race, admission year, primary payer status, socio-economic stratum, hospital characteristics, comorbidities, acute organ failure, cardiac procedures and non-cardiac procedures, was performed to identify predictors of AKI, hemodialysis use and in-hospital mortality. For the multivariate modeling, regression analysis with purposeful selection of statistically (p<0.20 by univariate analysis) and clinically relevant variables was conducted. Two-tailed p<0.05 was considered statistically significant. All analyses were performed using SPSS version 25.0 (IBM Corp, Armonk NY).

## Results

**Fig 1:**
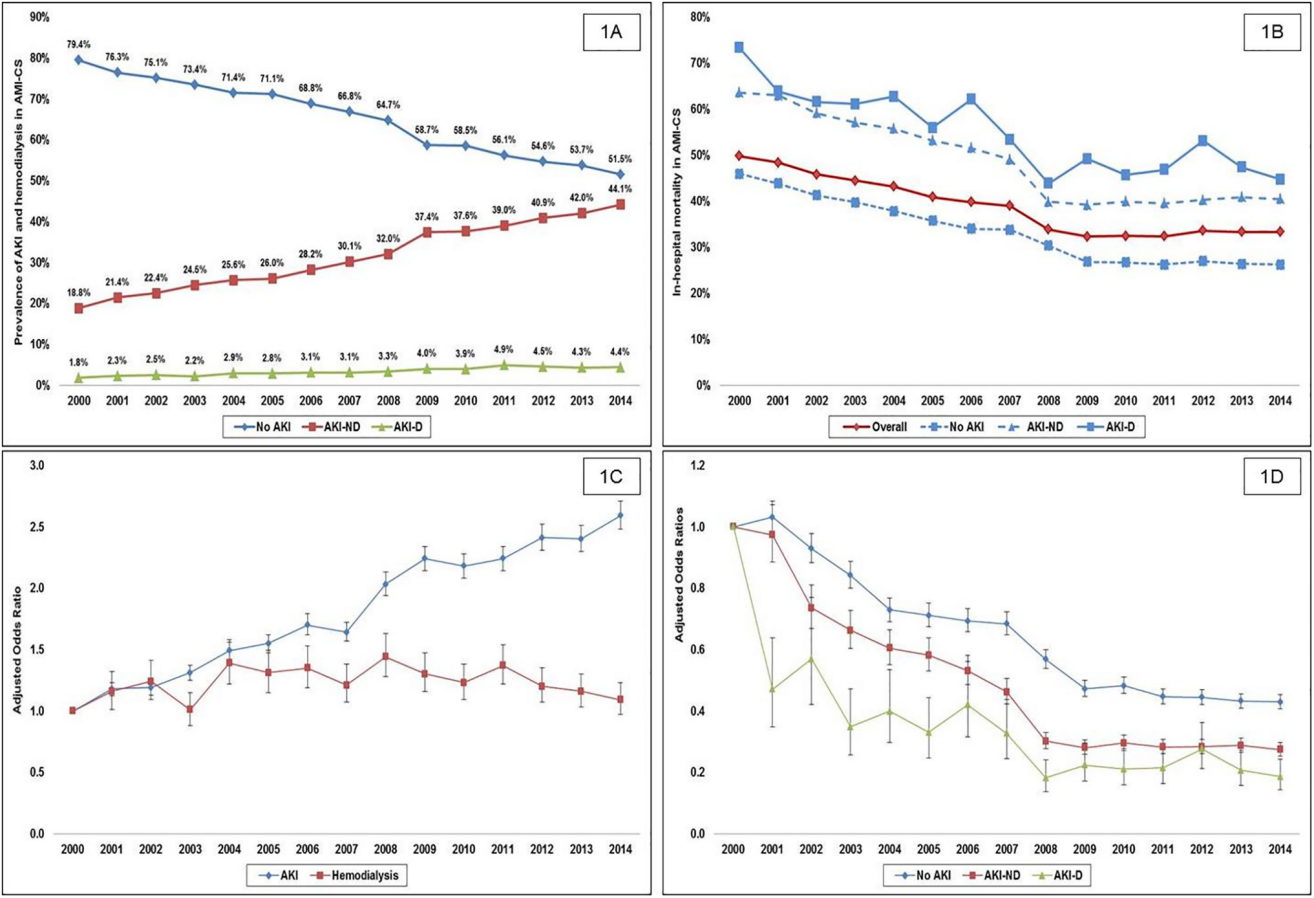
Unadjusted and adjusted temporal trends of the incidence and in-hospital mortality of acute kidney injury and hemodialysis in AMI-CS. 1A: Unadjusted fifteen-year temporal trends of no AKI, AKI-ND, AKI-D in AMI-CS; 1B: Unadjusted fifteen-year temporal trends of in-hospital mortality in the cohorts of no AKI, AKI-ND, AKI-D in AMI-CS; 1C: Adjusted temporal trends for the incidence of AKI, hemodialysis use in AMI-CS*; 1D: Adjusted temporal trends for in-hospital mortality in cohorts with no AKI, AKI-ND and AKI-D in AMI-CS*; all p<0.001 for trend. *Adjusted for: age, sex, race, primary payer, socio-economic status, hospital location/teaching status, hospital bedsize, hospital region, comorbidity, acute organ dysfunction, cardiac arrest, use of coronary angiography, percutaneous coronary intervention, invasive hemodynamic assessment, mechanical circulatory support and invasive mechanical ventilation. **Abbreviations:** AKI: acute kidney injury; AKI-D: acute kidney injury requiring hemodialysis; AKI-ND: acute kidney injury with no need for hemodialysis; AMI: acute myocardial infarction; CS: cardiogenic shock.

During this 15-year period, there were an estimated 444,253 admissions for AMI-CS. In the final cohort of 440,257 admissions after excluding admissions with prior hemodialysis, AKI was noted in 155,610 (35.3%), with AKI-D in 14,950 (3.4%). Compared to admissions without AKI, those with AKI-ND and AKI-D were more likely to be older (68±13, 71±13 and 69±12 years), male (59%, 64% and 66%), with higher rates of diabetes (4%, 5% and 5%), heart failure (50%, 64% and 70%), chronic kidney disease (7%, 23% and 36%), presenting with non-ST-elevation AMI-CS (27%, 40% and 47%), and received less frequent coronary angiography (70%, 63% and 65%) and percutaneous coronary intervention (51%, 42% and 37%) (all p<0.001). AKI admissions had higher rates of concomitant non-cardiac organ failure, cardiac arrest, and use of invasive hemodynamic monitoring, mechanical ventilation, and mechanical circulatory support (Table 1). Between 2000 and 2014, there was a steady increase in the unadjusted and adjusted incidence of AKI and AKI-D (Fig 1A and 1C). A multivariable regression model for significant predictors of AKI and AKI-D are presented in Table 2.

**Table 1:** Organ failure and management of AMI-CS with and without acute kidney injury.

Characteristic		No AKI (N = 284,647)	AKI-ND (N = 140,659)	AKI-D (N = 14,950)	P
**Acute organ failure**	**Respiratory**	36.5	55.0	65.0	<0.001
	**Hepatic**	3.1	15.6	26.9	<0.001
	**Hematological**	8.0	15.2	27.1	<0.001
	**Metabolic**	10.6	27.0	35.4	<0.001
	**Neurological**	10.1	18.7	22.6	<0.001
**Cardiac arrest**		16.9	20.0	20.8	<0.001
Invasive hemodynamic assessment*		18.6	21.9	28.1	<0.001
**Mechanical circulatory support**	**Total**	45.5	44.3	48.2	<0.001
	**IABP**	44.6	42.4	44.8	<0.001
	**Percutaneous MCS**	0.9	2.1	2.9	<0.001
	**Non-percutaneous MCS**	0.4	0.7	1.1	<0.001
	**ECMO**	0.3	0.9	1.8	<0.001
**Non-invasive ventilation**		2.3	4.3	5.6	<0.001
**Invasive mechanical ventilation**		36.5	50.2	69.5	<0.001

Represented as percentage;

*pulmonary artery catheterization or right heart catheterization

**Abbreviations:** AKI: acute kidney injury; AKI-D: acute kidney injury requiring hemodialysis; AKI-ND: acute kidney injury with no need for hemodialysis; AMI: acute myocardial infarction; CS: cardiogenic shock; ECMO: extracorporeal membrane oxygenation; IABP: intra-aortic balloon pump; MCS: mechanical circulatory support

**Table 2:** Multivariable regression for predictors of AKI and hemodialysis use in AMI-CS.

Total cohort (N = 440,257)		Acute kidney injury				Hemodialysis Use			
		OR	95 CI		P	OR	95 CI		P
			LL	UL			LL	UL	
**Age group (years)**	**19–49**	Reference category				Reference category			
	**50–59**	1.07	1.03	1.11	<0.001	1.03	0.94	1.13	0.47
	**60–69**	1.07	1.02	1.11	0.002	0.80	0.73	0.88	<0.001
	**70–79**	1.20	1.15	1.25	<0.001	0.79	0.71	0.88	<0.001
	**≥80**	1.26	1.20	1.32	<0.001	0.51	0.45	0.56	<0.001
**Female sex**		0.73	0.72	0.74	<0.001	0.88	0.85	0.92	<0.001
**Race**	**White**	Reference category				Reference category			
	**Black**	1.29	1.25	1.33	<0.001	1.25	1.17	1.34	<0.001
	**Hispanic**	1.10	1.07	1.13	<0.001	1.43	1.35	1.52	<0.001
	**Asian**	1.13	1.08	1.18	<0.001	1.38	1.26	1.51	<0.001
	**Native American**	0.97	0.87	1.08	0.57	1.54	1.24	1.91	<0.001
	**Others**	1.07	1.03	1.12	<0.001	1.32	1.21	1.44	<0.001
**Primary payer**	**Medicare**	Reference category				Reference category			
	**Medicaid**	1.08	1.04	1.12	<0.001	1.17	1.08	1.26	<0.001
	**Private**	0.93	0.91	0.95	<0.001	1.04	0.98	1.09	0.21
	**Uninsured**	0.86	0.82	0.89	<0.001	0.79	0.71	0.87	<0.001
	**No Charge**	1.13	1.01	1.26	0.03	1.36	1.07	1.73	0.01
	**Others**	0.95	0.90	1.00	0.06	1.05	0.93	1.19	0.39
**Quartile of median household** **income**	**0-25**^**th**^	Reference category				Reference category			
	**26**^**th**^**-50**^**th**^	1.02	1.00	1.04	0.07	1.05	1.00	1.11	0.07
	**51**^**st**^**-75**^**th**^	1.02	1.00	1.05	0.04	1.02	0.97	1.08	0.37
	**75**^**th**^**-100**^**th**^	0.98	0.96	1.00	0.11	1.00	0.94	1.06	0.93
**Hospital teaching** **status and location**	**Rural**	Reference category				Reference category			
	**Urban Non-Teaching**	1.30	1.26	1.35	<0.001	1.39	1.25	1.54	<0.001
	**Urban Teaching**	1.59	1.54	1.64	<0.001	1.80	1.62	2.00	<0.001
**Hospital bed-size**	**Small**	Reference category				Reference category			
	**Medium**	0.97	0.94	1.00	0.05	1.03	0.94	1.11	0.55
	**Large**	1.08	1.05	1.11	<0.001	1.33	1.24	1.44	<0.001
**Hospital region**	**Northeast**	Reference category				Reference category			
	**Midwest**	0.95	0.93	0.98	<0.001	1.08	1.01	1.15	0.02
	**South**	0.99	0.97	1.02	0.57	0.89	0.85	0.94	<0.001
	**West**	0.98	0.96	1.00	0.10	1.33	1.25	1.41	<0.001
**Charlson Comorbidity Index**	**0–3**	Reference category				Reference category			
	**4–6**	1.80	1.75	1.85	<0.001	1.94	1.81	2.08	<0.001
	**≥ 7**	3.12	3.02	3.23	<0.001	3.89	3.60	4.21	<0.001
**Acute organ dysfunction**	**Respiratory**	1.55	1.52	1.58	<0.001	1.35	1.29	1.40	<0.001
	**Hepatic**	4.24	4.11	4.37	<0.001	2.71	2.59	2.83	<0.001
	**Hematologic**	1.53	1.49	1.57	<0.001	2.01	1.92	2.10	<0.001
	**Metabolic**	2.25	2.20	2.29	<0.001	1.75	1.68	1.83	<0.001
	**Neurologic**	1.39	1.36	1.42	<0.001	1.12	1.07	1.18	<0.001
**Cardiac arrest**		0.90	0.88	0.91	<0.001	0.84	0.81	0.88	<0.001
**Coronary angiography**		0.81	0.79	0.82	<0.001	1.01	0.96	1.06	0.75
**Percutaneous coronary intervention**		0.76	0.75	0.78	<0.001	0.70	0.67	0.73	<0.001
**Invasive hemodynamic assessment**		1.33	1.30	1.35	<0.001	1.31	1.26	1.37	<0.001
**Mechanical circulatory support**		1.08	1.06	1.10	<0.001	1.09	1.04	1.13	<0.001
**Invasive mechanical ventilation**		1.20	1.17	1.22	<0.001	1.97	1.89	2.06	<0.001

**Abbreviations:** AKI: acute kidney injury; AMI: acute myocardial infarction; CI: confidence interval; CS: cardiogenic shock; LL: lower limit; OR: odds ratio; UL: upper limit

Compared to those with no AKI, all-cause in-hospital mortality was higher in AKI-ND (46% vs. 34%; OR 1.67 [95% CI 1.65–1.69]; p<0.001) and AKI-D cohorts (53% vs. 34%; OR 2.16 [95% CI 2.09–2.23]; p<0.001). Unadjusted and adjusted temporal trends for the overall population and the AKI cohorts showed a significant decrease in in-hospital mortality during the study period (Fig 1B and 1D). Compared to admissions without AKI, those with AKI-ND and AKI-D had a longer hospital length of stay (8.8 ± 9.7 days, 11.9 ± 13.4 days and 17.6 ± 18.7 days), higher adjusted total hospitalization costs ($101,859 ± 116,204, $159,804 ± 190,766 and $265,875 ± 254,919) and were discharged home less frequently (49.2%, 27.7% and 19.1%) and needed skilled nursing facilities after discharge more frequently (22.6%, 42.8% and 52%) (all p<0.001). In a multivariate regression analysis, compared to the cohort with no AKI, AKI-ND (OR 1.35 [95% CI 1.32–1.37]; p<0.001) and AKI-D (OR 1.73 [95% CI 1.66–1.80]; p<0.001) were independently associated with higher in-hospital mortality (**Table B in** S1 File).

## Discussion

In this nationally-representative population of AMI-CS, we noted a steady increase in AKI-ND and AKI-D, with 2.6-fold higher adjusted risk of developing AKI in 2014 compared to 2000. Despite a temporal increase in AKI and AKI-D, there was a temporal decrease in in-hospital mortality in all three cohorts. Older age, black race, non-private insurance, higher comorbidity, organ failure, use of organ support systems and admission to a large urban hospital were associated with the development of AKI and AKI-D. AKI-ND and AKI-D were associated with 1.3 and 1.7-fold higher mortality and with higher resource utilization.

The results of this study are consistent with prior data on organ failure in AMI-CS.[24, 25] Using the HCUP-NIS database, we have previously demonstrated that single- and multi-organ failure is associated with higher in-hospital mortality and resource utilization in AMI-CS.[5] Furthermore, non-cardiac organ failure is associated with continued post-hospitalization resource utilization as noted in this study.[3–6, 9, 11] The lower rates of angiography and percutaneous coronary intervention in admissions with AKI in this study are consistent with prior real-world literature that reflects reluctance to perform angiography in higher risk cohorts despite robust guideline recommendations.[26] Due to the limitations of this database, we are unable to time the onset of AKI with respect to coronary angiography, and therefore, it is possible that the AKI served as both a barrier and consequence of angiography.

The results of our study are consistent with data from other non-AMI populations, which have demonstrated a steady increase in AKI prevalence.[17, 27, 28] In unselected AMI patients, Amin et al. noted a decrease in the incidence and in-hospital mortality from AKI in all AMI patients.[13] They postulated that this may be partly due to greater recognition and the effective use of preventive strategies.[13, 29] In contrast to the general AMI population, our study noted an increasing trend of AKI in AMI-CS during this study period. Though this could be due to increased awareness and more accurate coding of AKI, thought the concomitant increase in the use of hemodialysis refutes this hypothesis. As we note, the increase in AKI prevalence despite decrease in overall mortality during this period allude to increasing severity of illness.[5] Prior population studies confirmed these findings that undifferentiated shock and respiratory failure are associated with higher risk of AKI-D.[29] In a recent study from the HCUP-NIS over 6 years (2010–2015), Adegbala et al. looked at all CS from any etiology and illustrated the characteristics of AKI-D in this population.[19] This current study is significantly different from the work by Adegbala et al. in that it includes a larger cohort (2000–2014) that provides more reliable temporal trends. We only included admissions with a primary diagnosis of AMI. Typically, CS from end-stage heart failure is associated with higher filling pressures, lower ejection fractions but with lesser end-organ hypoperfusion that is likely related to the chronicity of the process.[30] Furthermore, our work demonstrates an incremental nature of AKI and hemodialysis that was not previously reported.

In this study, we noted non-cardiac organ failure, older age, non-White race and higher comorbidity to be significant predictors of developing AKI during AMI-CS.[31, 32] Despite an increase in AMI-CS prevalence during the study period as noted in our prior work,[5] we note an overall temporal decrease in in-hospital mortality independent of AKI. This may be attributable to the limitations in the contemporary prevention and management strategies for AKI in the intensive care unit and the lack of detailed pathophysiological understanding of cardiorenal interactions.[15, 18, 33–36] Indeed given the high resource utilization, the focus should shift to prevention of AKI through rigorous quality improvement, judicious use of vasoactive medications and fluids, prompt diuresis and prevention of nephrotoxicity.[15, 18, 34, 35, 37–40]

The costs associated with AKI are significantly higher than that noted in literature.[14] It is possible that the advent of newer mechanical circulatory support devices, need for multi-organ support, and the unpredictable course of AMI-CS which might limit prognostication could have contributed to these costs.[7–9, 22, 23, 37, 41] Therefore, a careful team-based approach integrating goals-of-care, defining futility, and exit strategy from temporary circulatory support is urgently needed in these patients to optimize resource utilization.[1, 9] Lastly, admissions with cardiac arrest developed less AKI and had a lower rate of dialysis used in this study contrary to prior literature.[42] We postulate that patients with cardiogenic shock combined with cardiac arrest likely had earlier in-hospital mortality preventing the development of AKI and greater use of end-of-life decisions.

This study has several limitations. It is possible that the greater incidence of AKI over the years is likely related to greater recognition and improved coding practices.[43] The ICD-9CM codes for AMI and CS have been previously validated that reduces the inherent errors in the study. The timing of AKI and treatment-limiting decisions could not be reliably identified in this hospital admissions database. It is possible that a minority of the included admissions had creatinine elevations without AKI (i.e. no tubular injury). It is possible that sensitive definitions of AKI and the use of hemodialysis at lower thresholds of acuity may contribute to the increase in the prevalence of AKI-ND and AKI-D. However the concomitant rise in other organ failure refutes this possibility.[5] Despite these limitations, this study addresses an important knowledge gap highlighting the epidemiology of AKI and hemodialysis in AMI-CS in a contemporary 15-year period.

## Conclusions

In this population of 440,257 AMI-CS admissions, we noted a steady increase in AKI-ND and AKI-D during the 15-year study period. Presence of AKI-ND and AKI-D was associated with a 1.3-f and 1.7-fold higher mortality and increased resource usage, emphasizing the need to develop strategies for early identification and prevention of AKI in this critically ill population.

## Supporting information

S1 File Table A. Administrative codes used for identification of diagnoses and procedures. Table B. Multivariable regression for in-hospital mortality in AMI-CS.
(DOCX)

## Abbreviations

AKI: acute kidney injury
AKI-D: acute kidney injury requiring dialysis
AKI-ND: acute kidney injury not requiring dialysis
AMI: acute myocardial infarction
ARF: acute renal failure
CI: confidence interval
CS: cardiogenic shock
HCUP: Healthcare Cost and Utilization Project
ICD-9CM: International Classification of Diseases 9 Clinical Modification
NIS: National Inpatient Sample
OR: odds ratio
