# Supplementary material for: Temporal trends, predictors, and outcomes of acute kidney injury and hemodialysis use in acute myocardial infarction-related cardiogenic shock
Source: PLoS One. 2019 Sep 18;14(9):e0222894. doi: 10.1371/journal.pone.0222894 (PMC6750602; doi:10.1371/journal.pone.0222894)
Supplement: S1 File — Table A. Administrative codes used for identification of diagnoses and procedures. Table B. Multivariable regression for in-hospital mortality in AMI-CS. (DOCX) [file pone.0222894.s001.docx]

**Table A. Administrative codes used for identification of diagnoses and procedures**

| **Comorbidity** | **ICD-9CM Codes** |
| --- | --- |
| Cardiac arrest | 427.5 |
| Coronary angiography | 36.06, 37.22, 37.23, 88.53-88.56 |
| Percutaneous coronary intervention | 00.66, 36.01, 36.02, 36.05, 36.07, 88.57 |
| Right heart catheterization | 37.21, 37.23 |
| Swan Ganz catheterization | 204 (Clinical Classification Software) |
| Intra-aortic balloon pump | 37.61 |
| Percutaneous MCS | 37.68 |
| Non-percutaneous MCS | 37.60, 37.62, 37.65 |
| Extra-corporeal membrane oxygenation | 39.65 |
| Invasive mechanical ventilation | 96.7, 96.70, 96.71, 96.72 |
| Non-invasive mechanical ventilation | 93.90 |
| Acute respiratory failure | 518.81, 518.85, 786.09, 799.1 |
| Acute hepatic failure | 570.x, 572.2, 573.3, 573.4 |
| Acute metabolic failure | 276.2 |
| Acute neurological failure | 293, 293.0, 293.1, 293.8, 293.81-293.84, 293.89, 293.9, 348.1, 780.01, 780.09, 89.14, 348.3, 348.30, 348.31, 348.39 |

**Abbreviations:** ICD-9CM: International Classification of Diseases, 9.0 Clinical Modification; MCS: mechanical circulatory support

**Table B. Multivariable regression for in-hospital mortality in AMI-CS**

| **Total cohort**  **(N = 440,257)** | | **Odds ratio** | **95% confidence interval** | | ***P*** |
| --- | --- | --- | --- | --- | --- |
|  |  |  | **Lower Limit** | **Upper Limit** |  |
| **Acute kidney injury** | **No AKI** | Reference category | | | |
|  | **AKI-ND** | 1.35 | 1.32 | 1.37 | <0.001 |
|  | **AKI-D** | 1.73 | 1.66 | 1.80 | <0.001 |
| **Age groups (years)** | **19-49** | Reference category | | | |
|  | **50-59** | 1.27 | 1.22 | 1.32 | <0.001 |
|  | **60-69** | 1.85 | 1.77 | 1.93 | <0.001 |
|  | **70-79** | 3.00 | 2.86 | 3.14 | <0.001 |
|  | **≥80** | 4.90 | 4.67 | 5.14 | <0.001 |
| **Female sex** | | 1.11 | 1.09 | 1.13 | <0.001 |
| **Race** | **White** | Reference category | | | |
|  | **Black** | 0.98 | 0.95 | 1.01 | 0.27 |
|  | **Hispanic** | 1.03 | 1.00 | 1.06 | 0.06 |
|  | **Asian** | 0.98 | 0.93 | 1.03 | 0.38 |
|  | **Native American** | 1.08 | 0.97 | 1.20 | 0.15 |
|  | **Others** | 1.00 | 0.96 | 1.04 | 0.90 |
| **Primary payer** | **Medicare** | Reference category | | | |
|  | **Medicaid** | 0.96 | 0.92 | 0.99 | 0.02 |
|  | **Private** | 0.78 | 0.76 | 0.79 | <0.001 |
|  | **Uninsured** | 1.34 | 1.29 | 1.39 | <0.001 |
|  | **No Charge** | 0.86 | 0.76 | 0.97 | 0.01 |
|  | **Others** | 0.87 | 0.82 | 0.92 | <0.001 |
| **Quartile of median household**  **income for zip code** | **0-25^th^** | Reference category | | | |
|  | **26^th^-50^th^** | 0.94 | 0.92 | 0.96 | <0.001 |
|  | **51^st^-75^th^** | 0.91 | 0.89 | 0.93 | <0.001 |
|  | **75^th^-100^th^** | 0.87 | 0.85 | 0.89 | <0.001 |
| **Hospital teaching**  **status and location** | **Rural** | Reference category | | | |
|  | **Urban Non-Teaching** | 0.96 | 0.93 | 0.99 | 0.01 |
|  | **Urban Teaching** | 0.99 | 0.96 | 1.02 | 0.40 |
| **Hospital bed-size** | **Small** | Reference category | | | |
|  | **Medium** | 1.00 | 0.97 | 1.03 | 0.89 |
|  | **Large** | 1.01 | 0.98 | 1.04 | 0.46 |
| **Hospital region** | **Northeast** | Reference category | | | |
|  | **Midwest** | 0.97 | 0.95 | 0.99 | 0.01 |
|  | **South** | 1.01 | 0.99 | 1.03 | 0.40 |
|  | **West** | 0.92 | 0.90 | 0.94 | <0.001 |
| **Charlson Comorbidity Index** | **0-3** | Reference category | | | |
|  | **4-6** | 0.82 | 0.80 | 0.85 | <0.001 |
|  | **≥ 7** | 0.77 | 0.74 | 0.79 | <0.001 |
| **Acute organ dysfunction** | **Respiratory** | 1.12 | 1.10 | 1.14 | <0.001 |
|  | **Hepatic** | 1.38 | 1.34 | 1.42 | <0.001 |
|  | **Hematologic** | 0.79 | 0.77 | 0.81 | <0.001 |
|  | **Neurologic** | 1.44 | 1.40 | 1.47 | <0.001 |
| **Cardiac arrest** | | 2.57 | 2.52 | 2.62 | <0.001 |
| **Coronary angiography** | | 0.47 | 0.46 | 0.48 | <0.001 |
| **Percutaneous coronary intervention** | | 0.80 | 0.78 | 0.81 | <0.001 |
| **Invasive hemodynamic assessment** | | 0.99 | 0.97 | 1.01 | 0.29 |
| **Mechanical circulatory support** | | 1.13 | 1.11 | 1.15 | <0.001 |
| **Invasive mechanical ventilation** | | 1.75 | 1.72 | 1.79 | <0.001 |

**Abbreviations:** AKI: acute kidney injury; AKI-D: acute kidney injury requiring hemodialysis; AKI-ND: acute kidney injury with no need for hemodialysis; AMI: acute myocardial infarction; CS: cardiogenic shock
